# Galectina-3 na Pericardite Constritiva Crônica: Informações Precisas para o Bom Médico

**DOI:** 10.36660/abc.20200163

**Published:** 2020-05-12

**Authors:** Wolney de Andrade Martins

**Affiliations:** 1 Universidade Federal Fluminense Departamento de Medicina Clínica Niterói RJ Brasil Universidade Federal Fluminense, Departamento de Medicina Clínica, Niterói, RJ – Brasil; 2 Complexo Hospitalar de Niterói Niterói RJ Brasil Complexo Hospitalar de Niterói, Niterói, RJ – Brasil

**Keywords:** Galectina-3, Biomarcadores, Pericardite Constritiva, Pericárdio, Inflamação, Cardiomiopatia Restritiva

A galectina-3 (Gal-3), que agora é conhecida como um novo biomarcador, percorreu o caminho científico rigoroso da sua descoberta até a validação. Estudos experimentais e clínicos descreveram sua elevação em diversas situações, como tumores, insuficiência renal e insuficiência cardíaca.^1^ Sua administração causou fibrose miocárdica e insuficiência cardíaca (IC). Sua supressão ou inibição genética impediu a fibrose e a remodelamento, ou seja, a relação de causa e efeito foi comprovada.^2^ Níveis elevados de Gal-3 mostram um pior prognóstico, pois preveem a morte súbita. A galectina-3 foi preditora independente no curto e médio prazo de internações e de mortalidade em pacientes com IC, principalmente naqueles com insuficiência cardíaca com fração de ejeção preservada (ICFEP).^3^

O biomarcador pode auxiliar o clínico em seus impasses diagnósticos, na aferição do prognóstico e até na orientação da terapia. A ICFEP é um exemplo em que toda ajuda é bem-vinda. Múltiplas comorbidades, quadros menos típicas, especialmente em idosos e obesos, podem ser confusas. A ICFEP é uma das situações em que a Gal-3 pode auxiliar muito na confirmação diagnóstica.^4^

Fernandes et al.,^5^ apresentam um estudo de caso-controle no qual compararam 33 pacientes com pericardite constritiva crônica (PCC), predominantemente idiopática, com voluntários saudáveis. A justificativa era que a fibrose presente na PCC elevava os níveis de Gal-3 e isso estava relacionado a alterações morfológicas e funcionais típicas da PCC. Houve confirmação do diagnóstico de PCC por métodos de imagem, ecocardiografia e ressonância cardíaca, além de cirúrgicos. Foi um estudo difícil de realizar e somente foi possível em um centro de referência. Os resultados foram negativos e há inúmeras explicações possíveis. Uma amostra seletiva de pacientes com PCC idiopática é indicada pelos autores. Sabemos que a pericardite tuberculosa, que é de suma importância em áreas onde a tuberculose é endêmica, tem um curso clínico mais grave, com uma evolução comum de fibrose e constrição. A própria Gal-3 tem limitações devido à sua não-especificidade. É encontrada em processos inflamatórios e fibróticos nos pulmões, rins, fígado, pâncreas, e em pacientes com câncer, entre outros.

Historicamente, buscou-se o diagnóstico diferencial entre pericardite constritiva e cardiomiopatias restritivas através de parâmetros clínicos e laboratoriais. É plausível supor que a Gal-3 deva ser maior na segunda situação clínica, devido à magnitude do envolvimento miocárdico e intersticial. Em teoria, a Gal-3 também poderia esclarecer qual extensão do quadro clínico é devida à disfunção miocárdica nas cardiomiopatias ou restrição diastólica na pericardite constritiva. Ou seja, ainda existem inúmeras perguntas sem respostas baseadas em evidências.^6^

Considerando um paciente com um quadro clínico de insuficiência ventricular direita ou na investigação de ascites e valores “normais” de Gal-3, a publicação de Fernandes et al.^5^ permite inferir pontos a favor do diagnóstico de PCC em detrimento de cardiomiopatias restritivas ou outras doenças.

O estudo de Fernandes et al.,^5^ nos trouxe a novidade da medida da Gal-3 em uma situação muito específica, como a PCC. Isso mostrou claramente que não houve aumento significativo da Gal-3 ou uma associação com parâmetros morfológicos ou funcionais. A qualidade da pesquisa de Fernandes et al., aqui publicada nos Arquivos Brasileiros de Cardiologia, reside não apenas em sua originalidade, mas também em seus critérios metodológicos e rigor em relação a suas conclusões. O presente estudo levanta novas questões. Haveria uma diferença entre as etiologias da PCC? Haveria aplicabilidade da Gal-3 na diferenciação com cardiomiopatias restritivas? E a utilidade das dosagens seriadas de Gal-3?

Parafraseando o Dr. Alan Maisel, um renomado pesquisador de biomarcadores em cardiologia, “o biomarcador tornará o médico ruim, pior e o bom médico, melhor”.^7^ Portanto, as informações agora incorporadas na literatura pelos autores serão muito úteis para nós, desde que utilizadas dentro de um sentido clínico crítico.
