# Supplementary material for: Galectina-3 na Pericardite Constritiva Crônica: Informações Precisas para o Bom Médico
Source: Arq Bras Cardiol. 2020 May 12;114(4):690–1. [Article in Portuguese] doi: 10.36660/abc.20200163 (PMC9744344; doi:10.36660/abc.20200163)
Supplement: Translation

# Translation: Galectin-3 in Chronic Constrictive Pericarditis: Accurate Information for the Good Doctor

**Authors:** Wolney de Andrade Martins

**Affiliations:** 1 Brazil Universidade Federal Fluminense, Departamento de Medicina Clínica, Niterói, RJ – Brazil; 2 Brazil Complexo Hospitalar de Niterói, Niterói, RJ – Brazil

**Keywords:** Galectin-3, Biomarkers, Pericarditis Constrictive, Cardiomyopathy, Restrictive, Pericardium, Inflammation, Cardiomyopathy, Restrictive

Galectin-3 (Gal-3), which is now known as a new biomarker, has traveled the rigorous scientific pathway from discovery to validation. Experimental and clinical studies described its elevation in several situations, such as tumors, renal failure and heart failure.^1^ Its administration caused myocardial fibrosis and heart failure (HF). Its genetic suppression or inhibition prevented fibrosis and remodeling, that is, the cause-and-effect relationship had been proven.^2^ Elevated levels of Gal-3 show a worse prognosis, as they predict sudden death. Galectin-3 was an independent predictor in the short and medium term of hospitalizations and of mortality in patients with HF, especially those with heart failure with preserved ejection fraction (HFpEF).^3^

The biomarker can assist the clinician in their diagnostic dilemmas, in assessing the prognosis and even guiding the therapy. The HFpEF is an example of condition where all help is welcome. Multiple comorbidities, less typical conditions, especially in the elderly and obese, can be confusing. HFpEF is one of the situations where Gal-3 can greatly assist in diagnostic confirmation.^4^

Fernandes et al.^5^ present a case-control study in which they compared 33 patients with chronic constrictive pericarditis (CCP), predominantly idiopathic, with healthy volunteers. The rationale was that the fibrosis present in the CCP raised the levels of Gal-3, and this was related to the morphological and functional changes typical of CCP. There was confirmation of the diagnosis of CCP by imaging methods, echocardiography and cardiac resonance, as well as surgical ones. It was a difficult study to carry out and only possible in a reference center. The results were negative and there is a plethora of possible explanations. A selective sample of patients with idiopathic CCP is indicated by the authors. We know that tuberculous pericarditis, which is of paramount importance in areas where tuberculosis is endemic, has a more severe clinical course, with a common evolution to fibrosis and constriction. Gal-3 itself has limitations due to its non-specificity. It is found in inflammatory and fibrotic processes in the lungs, kidneys, liver, pancreas, and in cancer patients, among others.

Historically, we sought to attain the differential diagnosis between constrictive pericarditis and restrictive cardiomyopathies through clinical and laboratory parameters. It is plausible to assume that Gal-3 should be higher in the second clinical situation, due to the magnitude of myocardial and interstitial involvement. Theoretically, Gal-3 could also provide us with how much the clinical picture is due to myocardial dysfunction in cardiomyopathies or diastolic restriction in constrictive pericarditis. That is, there are still countless questions without answers based on evidence.^6^

Considering a patient with a clinical picture of right ventricular failure or during the investigation of ascites and “normal” Gal-3 values, the publication by Fernandes et al.^5^ allows us to infer points in favor of the diagnosis of CCP to the detriment of restrictive cardiomyopathies or other diseases.

The study of Fernandes et al.^5^ brought us the novelty of Gal-3 measurement in a very specific situation such as the CCP. It clearly showed that there was no significant increase in Gal-3 or an association with morphological or functional parameters. The quality of the research of Fernandes et al., herein published in Arq Bras Cardiol., lies not only on its originality, but also on its methodological criteria and rigor regarding its conclusions. The present study raises new questions. Would there be a difference between the CCP etiologies? Would there be any applicability of Gal-3 in differentiation with restrictive cardiomyopathies? What about the usefulness of serial measurements of Gal-3?

To paraphrase Dr. Alan Maisel, a renowned resercher of biomarkers in cardiology, “the biomarker will make the bad doctor worse and the good doctor better”.^7^ Therefore, the information now incorporated into the literature by the authors will be very useful to us, provided it is used within a critical clinical sense.
